# Next-generation vaccines against bacterial pathogens: mRNA and beyond

**DOI:** 10.3389/fimmu.2025.1709794

**Published:** 2025-12-05

**Authors:** Huimin Chen, Ye Gu, Lei Song, Lihui Si

**Affiliations:** 1 Department of Pediatric Surgery, The Second Hospital of Jilin University, Changchun, China; 2 Department of Respiratory Medicine, Center for Pathogen Biology and Infectious Diseases, Jilin Provincial Key Laboratory for Individualized Diagnosis and Treatment of Pulmonary Diseases, The First Hospital of Jilin University, Changchun, China; 3 Department of Obstetrics and Gynecology, The Second Hospital of Jilin University, Changchun, China

**Keywords:** mRNA vaccines, antimicrobial resistance, ESKAPE pathogens, tuberculosis, DNA vaccines, nanoparticle

## Abstract

The global rise of multidrug-resistant (MDR) bacterial infections has exacerbated the need for effective vaccines to prevent these hard-to-treat pathogens. Traditional vaccine approaches have achieved tremendous successes but often fall short for pathogens like *Mycobacterium tuberculosis* (TB), which evades host immunity through complex mechanisms, and for multidrug-resistant ESKAPE bacteria, where antibiotic resistance and antigenic variability complicate effective vaccine development. The COVID-19 pandemic spurred unprecedented advances in vaccine technology – particularly mRNA vaccines – reviving interest in novel platforms for bacterial diseases. Here we review next-generation vaccine strategies, focusing on nucleic acid-based platforms such as mRNA, DNA, and self-amplifying RNA (saRNA), as well as viral vector vaccines. We also examine nanoparticle technologies that serve as delivery systems or adjuvant platforms across these approaches. We discuss the unique opportunities of mRNA vaccines to induce both robust antibody and T-cell responses required for intracellular infections like TB, as well as the challenges of antigen discovery and delivery (e.g. lipid nanoparticles). Each platform’s mechanism, immunogenic profile, current development status, and challenges are analyzed, including comparative insights. We highlight recent progress such as mRNA vaccine candidates against TB entering clinical trials and saRNA prototypes protecting against plague in animals. Finally, we provide a perspective on the future of vaccine strategies to combat antimicrobial resistance (AMR) – emphasizing the integration of multiple platforms, global collaborative efforts, regulatory pathways, and the translational hurdles that must be overcome to bring these next-generation vaccines from bench to bedside.

## Introduction

Antimicrobial-resistant bacterial infections are a critical and growing threat to global health. Multidrug-resistant (MDR) bacteria already cause millions of difficult-to-treat infections annually. For example, in the United States over 2 million antimicrobial resistance (AMR) infections occur each year, leading to approximately 29,000 deaths and over $4.7 billion in medical costs (1). In Europe, drug-resistant bacteria are implicated in more than 33,000 deaths per year (1). Without urgent action, the future impact is expected to be catastrophic – recent projections estimate about 39 million deaths attributable to bacterial AMR between 2025 and 2050 (roughly three deaths every minute) if current trends continue (2). The World Health Organization has recognized AMR as a major global health threat, calling for coordinated international efforts to curb its spread (3). Alongside the judicious use of antimicrobial agents and new drug development, preventive vaccines are a cornerstone in the fight against AMR by reducing infection incidence and thereby decreasing the need for antibiotics (2, 4). However, many of the most dangerous bacterial pathogens lack effective vaccines.

Traditional vaccine modalities – live-attenuated and inactivated whole-cell vaccines, toxoids, and subunit/protein vaccines – have limitations that hamper their utility against many AMR threats. Live-attenuated bacterial vaccines (such as BCG for TB or oral typhoid vaccine) can elicit strong immunity but may pose safety risks in immunocompromised recipients and can be difficult to develop for highly virulent organisms (5). Killed/inactivated vaccines are safer but often lose some immunogenicity during the inactivation process (5). Purified subunit or polysaccharide-protein conjugate vaccines (e.g. pneumococcal or meningococcal conjugate vaccines) are very safe, yet they generally require adjuvants and booster doses to achieve durable protection (6). Generating robust T-cell responses – crucial for defense against intracellular bacteria – is a particular challenge for subunit vaccines (7, 8). In summary, traditional platforms sometimes fail for pathogens that have complex antigenic profiles, high variability, or intracellular lifestyles. These shortcomings have spurred the search for novel vaccine technologies that can overcome the challenges of identifying protective bacterial antigens and eliciting robust, targeted immune responses.

A vaccine platform is a standardized, reusable technology stack (vector, process and analytics) into which new antigens can be inserted with minimal changes, allowing rapid product generation across pathogens. In recent years, next-generation vaccine platforms have rapidly advanced, holding promise to revolutionize bacterial vaccinology (4, 9–11). Nucleic acid-based vaccines and viral vaccines offer unprecedented flexibility and speed in design. In parallel, nanoparticle technologies serve as key delivery systems and adjuvant platforms that support these approaches. Notably, the COVID-19 pandemic catalyzed global proof-of-concept for some of these technologies. The first mRNA vaccines (for SARS-CoV-2) were developed, approved, and deployed at record speed, demonstrating the platform’s safety and efficacy in hundreds of millions of recipients. This success has initiated a new era in vaccine development and renewed hope for tackling diseases where conventional vaccines have failed. The pandemic also accelerated innovations in self-amplifying RNA vaccines (a related RNA platform) and viral vector vaccines (e.g. adenovirus-based COVID-19 vaccines), further validating their potential (12–14). These platforms offer advantages of rapid production, genetic flexibility, and induction of broad immune responses, which could be game-changing for bacterial vaccine targets (15). Indeed, mRNA vaccines are now being actively explored for bacterial infections, including difficult targets like TB and invasive *E. coli*, leveraging their ability to induce strong T-cell responses needed for intracellular pathogens (16).

**Figure 1:**
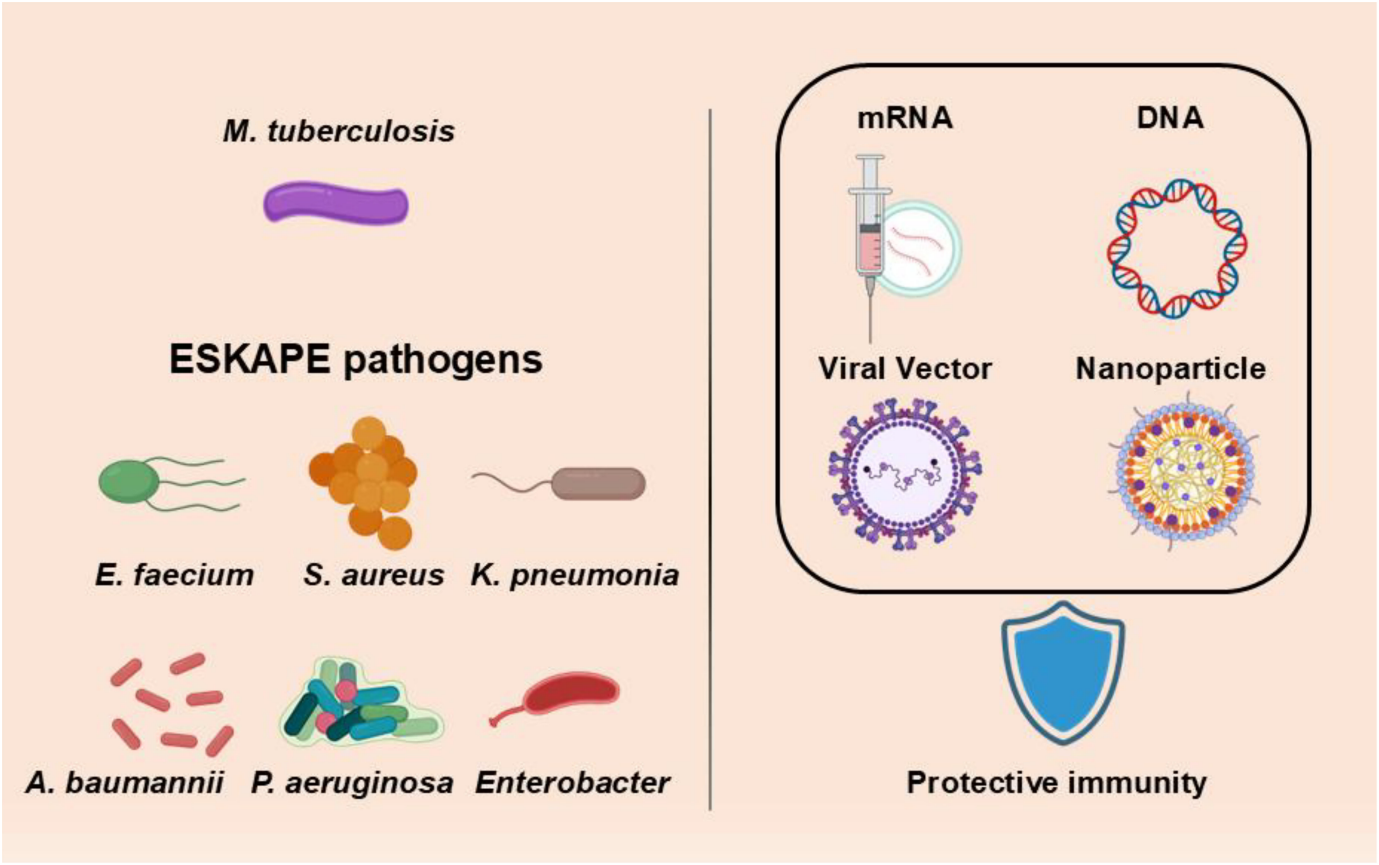
Schematic overview of major bacterial vaccine targets and novel platform strategies.

In the sections that follow, we review each next-generation vaccine platform in turn — including mRNA vaccines, self-amplifying RNA vaccines, DNA vaccines, and viral-vectored vaccines — followed by a dedicated discussion of nanoparticle technologies as delivery systems. We focus on their principles, current progress against bacterial pathogens, and the unique opportunities and challenges they present. A comparative summary of these platforms is provided (**Table 1**). Throughout, particular emphasis is placed on mRNA vaccines as the leading platform to emerge from the COVID-19 experience, and on their application to priority AMR pathogens such as the ESKAPE bacteria and *M. tuberculosis* (**Figure 1**). We discuss protective antigen discovery (e.g. using reverse vaccinology and machine learning to identify conserved bacterial targets), delivery systems like lipid nanoparticles (LNPs) that enable nucleic acid vaccine efficacy, and the status of preclinical models and clinical trials. Finally, we examine how these innovative platforms may be integrated into vaccine strategies to combat AMR, and what collaborative and regulatory efforts will be necessary to achieve widespread public health impact.

**Table 1:** Comparison of next-generation vaccine platforms for bacterial pathogens.

Vaccine Platform	Mechanism of Action	Key Advantages	Challenges	Examples / Status
mRNA vaccines	mRNA encoding a bacterial antigen is delivered (e.g. via LNP) into host cells, which translate it into antigen protein that triggers immune responses.	- Rapid design and production from sequence data (very adaptable to emerging strains). Induces both strong antibody and T-cell responses (antigen expressed inside cells stimulates MHC I and II pathways). No live pathogen or replication involved (non-infectious, safe even for immunocompromised). Can include multiple mRNA sequences in one vaccine (multivalent targeting of several antigens).	- mRNA is inherently unstable and requires cold-chain storage (though improvements are ongoing). Delivery requires efficient vectors (LNPs); LNPs can cause reactogenicity (injection site pain, fever) due to innate immune activation. No licensed mRNA vaccine for bacteria yet; regulatory experience mostly with viral vaccines. Scale-up manufacturing is costly, though costs are falling with technology advances.	COVID-19 mRNA vaccines validated platform in humans. Multiple preclinical bacterial mRNA vaccines in development (e.g. *M. tuberculosis* mRNA vaccine in Phase I trial). Plague mRNA (saRNA) vaccine protected mice.
Self-amplifying RNA (saRNA)	Similar to mRNA but the RNA includes viral replicase genes, so after delivery it self-replicates in cells, amplifying the antigen expression.	- Greatly reduced dose needed due to RNA amplification in vivo (dose-sparing). Prolonged antigen production can improve immune response magnitude and duration. Shares mRNA advantages: rapid design, induces broad immunity, non-infectious. First saRNA vaccine approved in 2023 (for COVID-19) proved feasibility.	- Much larger RNA molecule (replicon ~9–10 kb) can be harder to deliver (packaging into LNPs, cellular uptake efficiency). Can trigger stronger innate responses; need to modulate reactogenicity (excessive interferon may dampen antigen translation). Limited clinical data so far beyond COVID; no bacterial saRNA vaccines in trials yet.	saRNA COVID-19 vaccine (e.g. Arcturus) approved– showed equivalent immunity at fraction of mRNA dose. Plague saRNA vaccine (F1 + V antigens) protected mice at 1 µg dose. Being explored for TB and other infections requiring T<sub>H</sub>1 responses.
DNA vaccines	Plasmid DNA encoding antigen is injected into host tissue; cells take up the DNA, antigen is expressed inside cells (requires nuclear entry for transcription).	- Highly stable (plasmid DNA is robust at room temp, simplifying distribution). Easy and inexpensive to manufacture in bulk (bacterial fermentation). No risk of vector immunity; can be given repeatedly. Induces both humoral and cellular immunity if delivered efficiently (antigen expressed intracellularly like mRNA). Can encode multiple antigens in one plasmid (or co-administer multiple plasmids) for broad coverage.	- Poor natural uptake by human cells – delivery is the biggest hurdle (needs electroporation or special devices). Initial human trials showed low immunogenicity without advanced delivery methods. Requires nuclear entry for expression, which is inefficient in non-dividing cells (like muscle). Theoretical integration risk, though very low, necessitates safety monitoring. Few DNA vaccines have reached late-stage trials for bacteria (perceived as less “proven” than viral vectors or mRNA currently).	Several veterinary DNA vaccines licensed (e.g. West Nile in horses). COVID-19 DNA vaccine (ZyCoV-D) approved in 2021, delivered via needle-free injector. Preclinical successes for bacteria: e.g. DNA vaccine against *K. pneumoniae* OMPs protected mice; DNA vaccines for TB showed efficacy in animal models. Ongoing R&D with improved delivery (electroporation, nanocarriers) to enter human trials for TB, *C. diff*, etc.
Viral vector vaccines	A harmless viral vector (e.g. adenovirus, MVA poxvirus) is engineered to carry a gene for a bacterial antigen. The vector infects host cells and produces the antigen internally, eliciting immune responses.	- Very immunogenic: strong T-cell responses (especially CD8+) due to intracellular antigen expression and potent innate sensing of the vector. Often effective with a single dose (e.g. J&J’s Ad26 COVID vaccine), which is useful in outbreak control. Can target mucosal immunity by using vectors that infect via mucosal routes (e.g. intranasal adenovirus for respiratory pathogens). Track record of licensed products (Ebola, COVID vectors) gives regulatory and manufacturing know-how.	- Anti-vector immunity can reduce efficacy: prior exposure to common vectors (Ad5, etc.) or antibodies developed after first dose may blunt booster doses. Safety concerns: rare adverse events (e.g. clotting issues with adenoviral COVID vaccines) necessitate caution. Live viral vectors contraindicated in certain groups (e.g. severe immunosuppression) depending on vector type. More complex to manufacture (need cell culture to grow vectors, stringent bioengineering). Typically limited number of antigens can be delivered per vector (though large vectors like pox can carry multiple genes).	Multiple TB vaccine candidates use viral vectors: MVA85A (MVA vector) and Ad85A (adenovirus) have been tested in humans, showing robust immune responses. Adenovirus and chimp adenovirus vectors explored for *S. aureus* and *Pseudomonas* vaccines in preclinical models. Licensed viral vectored vaccines (not for bacteria yet) demonstrate platform potential – e.g. VSV-vectored Ebola vaccine, Ad26 COVID-19 vaccine. Prime-boost regimens (e.g. ChAdOx1 prime, MVA boost for TB) are in trials to overcome vector immunity and maximize responses.
Nanoparticle vaccines	Antigen is delivered on or within nanoparticles (~20–200 nm) made of proteins, lipids, or polymers. Examples: protein self-assembling nanoparticles displaying antigen; polymer NP encapsulating antigen and adjuvant; outer membrane vesicles from bacteria.	- **Multivalent display**: present repetitive antigen arrays that strongly activate B cells (mimicking pathogen surface). Can induce robust humoral and cellular immunity; efficiently taken up by dendritic cells, enhancing T-cell priming. Customizable co-delivery of adjuvants for tailored immune profiles (Th1 vs Th2, etc.). Flexible design: can include antigens from multiple pathogens in one particle; or target conserved epitopes from variants. Generally good stability; many nanoparticles are biodegradable with safe profiles.	- Manufacturing complexity: ensuring consistent NP size, antigen loading, and batch-to-batch reproducibility is challenging. Novel materials may face regulatory hurdles (each new nanoparticle might require extensive safety validation). Some NP formulations might induce unwanted inflammation if not properly engineered (need to balance immunogenicity and reactogenicity). Cost of goods could be higher for complex nanoparticles (depending on materials like recombinant proteins, etc.). Limited human data for bacterial nanoparticle vaccines so far (though several viral nanoparticle vaccines exist).	Licensed examples: OMV-based MenB vaccine (Bexsero) – an OMV is a natural nanoparticle from *N. meningitidis*. Multiple nanoparticle vaccines in development for AMR pathogens: e.g. gonococcal OMV vaccine, staphylococcal toxoid nanoparticles, and ferritin nanoparticle with *S. aureus* antigens. Peptide nanofiber vaccine for TB induced strong Th1 in mice. Nanoparticle adjuvants (like liposomal MPL in AS01) already used in vaccines – paving way for more NP-based delivery.

This table summarizes key features, advantages, and challenges of mRNA vaccines, self-amplifying RNA vaccines, DNA vaccines, viral vector vaccines, and nanoparticle vaccines in the context of developing immunizations against bacterial diseases.

## Nucleic acid-based vaccine platforms

### mRNA vaccines

Messenger RNA (mRNA) vaccine technology has rapidly advanced into a leading platform owing to its ability to induce strong immune responses, its speed of development, and its adaptability across various pathogens. mRNA vaccines work by delivering a piece of *in vitro*-transcribed mRNA encoding a target antigen; when taken up by host cells, the mRNA is translated into the antigen protein, which then triggers an immune response. This approach effectively turns the host’s cells into “vaccine factories” for the antigen, obviating the need to culture pathogens or to produce protein subunits externally. The result is a potent activation of immunity: mRNA vaccines are highly immunogenic, stimulating both B-cell mediated antibody production and CD8+ cytotoxic T lymphocyte responses (17, 18). This capability is particularly important for bacterial pathogens that require strong cell-mediated immunity for protection (e.g. intracellular organisms like *M. tuberculosis*). Notably, mRNA vaccines have an inherent adjuvant effect as well – the RNA molecules can activate innate immune sensors (such as endosomal Toll-like receptors) that promote dendritic cell maturation and robust immune activation (19). These properties differentiate mRNA vaccines from traditional protein vaccines, which often primarily induce antibody responses and typically require separate adjuvants.

Rapid design and production are major advantages of the mRNA platform. Once a target antigen’s genetic sequence is known, a corresponding mRNA vaccine can be synthesized in a matter of weeks, as demonstrated during COVID-19 (20). For rapidly evolving bacteria or outbreak scenarios, this speed is transformative. mRNA vaccines can also be rapidly adapted to new strains or variants: the nucleotide sequence can be modified as needed to match emerging mutations in bacterial virulence factors or resistance genes (1). This agility is valuable for highly mutable organisms (such as *Pseudomonas aeruginosa* or *Staphylococcus aureus*) that can quickly evolve to evade immune pressure (1). Additionally, unlike live-attenuated or inactivated vaccines which often focus on a whole organism, mRNA vaccines permit precise targeting of specific antigens – including multiple antigens at once. It is feasible to combine several mRNAs in one formulation to encode a cocktail of proteins, thereby addressing the antigenic complexity of many bacteria. For example, one can envision an mRNA vaccine targeting several conserved virulence factors of *Pseudomonas aeruginosa* simultaneously (e.g. PcrV plus flagellin components), a strategy shown to improve protection in animal models (21, 22). This multi-antigen design is a promising approach to broaden coverage and prevent immune escape. Delivery systems are central to mRNA efficacy. mRNA is a large, negatively charged molecule that would be degraded by RNases or fail to enter cells without a carrier. Clinically, lipid nanoparticles (LNPs) are the proven delivery vehicle: they protect mRNA from degradation and facilitate uptake and endosomal release into host cells (23, 24). Advances in LNP chemistry (such as ionizable lipids) have been pivotal, and LNPs themselves help direct mRNA to antigen-presenting dendritic cells within lymph nodes, where they initiate and enhance immune activation (25).

Although no bacterial mRNA vaccine is yet licensed, progress is promising. In tuberculosis (TB), BioNTech has developed multi-antigen mRNA candidates (BNT164) that recently entered Phase I trials (26, 27). These aim to induce strong TH1-type cellular immunity and IFN-γ production to improve on BCG’s performance (27). Several efforts also target ESKAPE pathogens with mRNA. For example, researchers are testing mRNAs encoding toxins or surface proteins of *Staphylococcus aureus*, *P. aeruginosa*, and others, often achieving protective immunity in animals (21, 28–31). mRNA vaccines also face specific challenges with bacteria. Antigen selection is complex due to bacterial genomic complexity and their ability to shift between physiological states (e.g., dormant, intracellular, biofilm-associated). Protective immunity often requires targeting multiple antigens or conserved bacterial components, such as virulence factors or essential surface proteins. In silico tools (reverse vaccinology, machine learning) are being used to predict promising antigens, but candidates still need careful validation in models. Bacteria can evade immunity by varying surface molecules or forming biofilms, so chosen antigens should ideally be invariable or essential. Another issue is the required response magnitude: some bacteria cause disease with very low inocula, so vaccines must induce high and durable antibody titers or T-cell responses, potentially using optimized doses or prime-boost regimens (32).

mRNA-LNP vaccines can cause transient systemic reactions (fever, injection site inflammation) due to innate immune sensing; these are usually short-lived and manageable (33). Ongoing work is optimizing LNP formulations to reduce reactogenicity while maintaining immunogenicity (33). Another concern is stability: first-generation mRNA vaccines required ultracold storage, which is challenging for wide distribution. Newer formulations and lyophilized mRNA are improving thermostability, but ensuring cold-chain logistics remains important for deployment in resource-limited regions. Cost is also a factor – mRNA production is currently expensive – but economies of scale and manufacturing advances are steadily lowering cost-per-dose. Despite these hurdles, mRNA’s flexibility and potency position it as a lead platform for difficult pathogens. Its ability to induce balanced humoral and cellular immunity to precisely defined antigens is a game-changer for diseases like TB and antimicrobial-resistant (AMR) bacteria. As mRNA vaccine research expands beyond viruses into bacterial diseases, experience will accumulate on how to tailor antigen selection, delivery, and dosing for each pathogen context.

### Self-amplifying RNA vaccines

Self-amplifying RNA (saRNA) vaccines are modified mRNAs that include viral replicase genes along with the antigen gene (34). Once inside a cell, the saRNA’s replicase proteins amplify the antigen-encoding RNA, producing many copies from a tiny initial dose (34). This greatly increases antigen production: studies have shown saRNA can achieve immune responses comparable to conventional mRNA using only a fraction (often 1/10th to 1/100th) of the dose (35, 36). In preclinical models, saRNA’s dose-sparing effect is striking. For example, a 1 µg dose of plague saRNA protected most mice from lethal *Y. pestis* challenge (37). Similar dose-sparing has been observed in other systems, suggesting saRNA could be valuable when rapid, large-scale vaccination is needed. In 2023, the first saRNA vaccine (for COVID-19) was approved, demonstrating that the platform can meet regulatory standards for safety and efficacy (35). Multiple saRNA vaccines are now in trials, which paves the way for applying the technology to bacterial targets.

Mechanistically, saRNA retains mRNA’s key advantages: no live pathogen, no genomic integration, and rapid design from sequence data. However, the viral replicase can itself activate innate immunity, which is a double-edged sword: mild innate activation can boost vaccine efficacy, but excessive interferon responses may suppress antigen expression. Early saRNA designs encountered this issue, so modern constructs are engineered to balance replication efficiency and innate sensing (38). However, a challenge is saRNA’s large size (~9–10 kilobases), which can complicate LNP encapsulation and uptake. Recent work shows LNPs can accommodate these large RNAs, and manufacturing processes are adapting, but efficient delivery of saRNA remains an area of optimization. Like mRNAs, saRNAs currently require low-temperature storage, although efforts to improve thermostability are ongoing.

saRNA vaccines use non-replicating replicons, meaning they cannot generate infectious virus, and thus retain a safety profile similar to that of mRNA vaccines. Early human trials of saRNA vaccines have reported acceptable safety, with side effects similar to mRNA at vastly lower doses (39). This is encouraging for future use in large populations.

Looking ahead, mRNA and saRNA are complementary. Standard mRNA is simpler to make, while saRNA offers extreme dose-sparing. In a sudden bacterial outbreak, one could envision priming with saRNA (for a strong cellular response with minimal material) followed by an mRNA boost (to fine-tune antibody responses). The optimal choice will depend on the pathogen’s characteristics. In sum, saRNA extends RNA vaccine utility by amplifying antigen expression and immunogenicity from less input material – a highly promising proposition for combating AMR pathogens on a large scale.

### DNA vaccines

DNA vaccines were among the first nucleic-acid platforms explored. A DNA vaccine uses a circular plasmid encoding one or more bacterial antigens under a eukaryotic promoter (40). After injection (often intramuscularly), cells take up the plasmid, transcribe it into mRNA in the nucleus, and translate the antigen protein. Like mRNA vaccines, DNA vaccines can elicit both antibodies and T-cell responses against the encoded antigens. Key advantages of DNA vaccines are their simplicity and stability: plasmids are easy to manufacture in bulk by bacterial fermentation and are extremely stable at room temperature (requiring no cold chain) (41). DNA vaccines also avoid anti-vector immunity (the plasmid itself is non-immunogenic as a carrier) and are considered very safe (non-replicating and minimal risk of genome integration) (41).

Initially, DNA vaccines showed great promise in animal studies, but early human trials were disappointing (42). Unassisted (“naked”) DNA delivery in humans led to very low antigen expression and weak immune responses, because plasmid uptake by adult muscle or skin cells is inefficient (43). Interest waned until new delivery methods were developed. Electroporation (applying an electrical pulse at the injection site) dramatically increases DNA uptake and protein expression (44). Other approaches include gene-gun delivery (DNA-coated particles) and advanced formulations (e.g. DNA-lipid complexes) (45, 46). With these improvements, some DNA vaccines have succeeded: notably, a DNA COVID-19 vaccine (ZyCoV-D) was approved in India in 2021 using a needle-free injector (47). Veterinary DNA vaccines have also been licensed, confirming that with proper delivery DNA can be effective (48).

For bacterial diseases, DNA vaccines have shown encouraging results in animals. Plasmids encoding key antigens from pathogens like *Klebsiella pneumoniae*, *S. aureus*, or *M. tuberculosis* have induced protective immunity in mice (49–51). DNA vaccines are well suited to multivalent designs: a single plasmid can carry multiple antigen genes, or multiple plasmids can be mixed in one shot, allowing a single vaccine to target many bacterial virulence factors at once (10).

To enhance DNA vaccine efficacy, strategies include built-in adjuvants and prime-boost regimens. Many plasmids contain unmethylated CpG motifs that activate innate immunity (TLR9), giving an intrinsic adjuvant effect (52). Researchers have co-delivered plasmids encoding cytokines (e.g. IL-12) to skew responses toward Th1, which is crucial for intracellular pathogens (53). Heterologous prime-boost regimens are also explored: for instance, a DNA prime followed by a viral-vector or protein boost has shown synergistic enhancement of T-cell and antibody responses in TB studies (54).

In summary, DNA vaccines remain a promising and versatile platform for bacterial immunization. Their robustness and low cost make them attractive for global deployment, especially in resource-limited settings. The main hurdle has been delivery and immunogenicity in humans, but advanced methods like electroporation and nano-formulations are overcoming this. As these technologies mature, DNA vaccines may finally realize their potential in preventing AMR infections. DNA and mRNA vaccines share the fundamental principle of delivering genetic instructions, yet each has practical strengths, and together they expand our toolkit against bacterial pathogens.

## Delivery technologies for bacterial vaccines

Nanoparticle vaccines present antigens on or within engineered nanoscale particles to enhance immune activation (55). In this review, mRNA/saRNA vaccines formulated in lipid nanoparticles (LNPs) are treated as nucleic-acid vaccine platforms using NP delivery technologies, not as “nanoparticle vaccines.” The term “nanoparticle vaccines” is reserved for antigen-displaying nanoparticle formulations (e.g., VLPs, OMVs, protein nanoparticles, liposomes with surface-displayed antigens) (55). The unifying principle is multivalent antigen display: nanoparticles can show many copies of an antigen in dense, repetitive arrays, efficiently cross-linking B-cell receptors and greatly boosting antibody responses (56). They can also co-deliver adjuvants and target antigen-presenting cells (APCs), improving both humoral and cellular immunity (56).

Several NP platforms are under investigation for bacterial vaccines:

- VLPs/OMVs: Self-assembled protein shells (VLPs) or naturally shed bacterial membrane vesicles (OMVs) displaying antigens. A licensed example is the *Neisseria meningitidis* B vaccine (Bexsero), which uses *N. meningitidis* OMVs (57). Researchers are now engineering OMVs from ESKAPE pathogens (detoxified and loaded with conserved antigens) to create broad “nanovaccines” against Gram-negative hospital bacteria (58).
- Protein Nanoparticles: Scaffold proteins (e.g. ferritin) form cage-like NPs displaying fused bacterial epitopes. This multivalent display markedly raises antibody responses. For instance, attaching pneumococcal polysaccharides or Group A *Streptococcus* M-protein peptides to protein NPs generated much stronger neutralizing antibodies than soluble vaccines (59).
- Polymer NPs: Biodegradable polymers (such as PLGA) can encapsulate antigen and adjuvant together. Upon uptake by dendritic cells, these NPs release cargo intracellularly, driving both MHC-II (CD4^+^ T-cell) and cross-presentation MHC-I (CD8^+^ T-cell) pathways (60). For example, mannan-decorated PLGA nanoparticles delivering a multi-epitope *M. tuberculosis* antigen effectively target dendritic cells, enhancing Th1/Th17 immune responses and providing protective efficacy against tuberculosis in mice (61). By adjusting polymer composition, release rates and antigen stability can be tuned.
- Lipid NPs/Subunit Carriers: Similar to mRNA LNPs, cationic liposomes can carry protein antigens or peptides. The AS01 adjuvant in Shingrix is a liposomal NP with immune stimulants (62). Displaying bacterial antigens on liposomes in a multivalent/arrayed format can enhance antibody induction; for example, liposome-displayed *enterotoxigenic Escherichia coli* (ETEC) colonization antigens elicited robust functional antibody responses (63).
- Nanotoxoids: When detoxified and encapsulated in nanoparticles, bacterial toxins can function as toxoid-like immunogens: the NP formulation abrogates cytotoxicity while preserving conformational epitopes, thereby eliciting neutralizing antibody responses without an additional carrier (64). This approach, applied to *S. aureus* alpha-hemolysin, protected animals from lethal toxin challenge (65).

Challenges remain for nanoparticle vaccines. Manufacturing consistency (uniform particle size, antigen loading) and scalability can be difficult (66). Regulatory pathways for novel nanomaterials are still evolving; each new NP formulation often requires extensive safety evaluation. Biocompatibility is generally addressed by using biodegradable materials (e.g. PLGA), but any persistent inorganic NP would raise safety questions (66). Stability varies: many polymer and lipid NPs can be lyophilized or refrigerated, but some may still need careful handling. There is also a theoretical risk that overly strong NP-induced immunity could cause immunopathology, so formulations must balance potency with safety.

Nonetheless, nanoparticle vaccines have proven platforms. Licensed NP-based vaccines (e.g. the AS01 adjuvant in Shingrix and VLP vaccines for HPV/hepatitis B) demonstrate that this approach can meet safety and efficacy standards. For AMR bacteria, many NP vaccine candidates are in the pipeline. In summary, nanoparticle vaccines offer a flexible, potent strategy: by co-delivering multiple antigens and adjuvants on one particle, a single formulation could neutralize several virulence mechanisms of an AMR pathogen. As research progresses, more NP-based bacterial vaccines are expected to enter clinical trials, bringing this transformative approach closer to reality.

## Viral vector vaccines

Viral vector vaccines use harmless, engineered viruses (such as adenoviruses or poxviruses) to deliver microorganisms antigen genes into host cells (67). The vector infects cells and produces the antigen internally, eliciting a potent immune response to that antigen. This strategy activates innate immune pathways similar to those triggered during viral infections, thereby providing built-in adjuvant signals. Viral vectors have a strong track record: adenoviral and poxviral vectors have been widely used in vaccines for Ebola and COVID-19, demonstrating that they can induce robust immunity even after a single dose (68, 69). Viral vectors are particularly valuable against pathogens requiring strong cellular immunity. Multiple TB vaccine candidates use viral vectors as booster immunizations. For example, MVA85A (an MVA poxvirus expressing the TB antigen 85A) safely boosted TB-specific T cells in humans (70). Adenovirus vectors encoding TB antigens (Ag85, TB10.4, etc.) have shown strong immunogenicity in animal studies and advanced to clinical trials (71). Beyond TB, viral vectors are being studied for other bacteria. In mice, adenovirus vectors expressing *S. aureus* toxin or adhesion genes elicited neutralizing antibodies and T cells that reduced bacterial load (72). An intravenously or intramuscularly injected adenovirus carrying *C. difficile* toxin fragments induced protective systemic antibodies (73, 74). Researchers have also tested vectors for *P. aeruginosa* and *Chlamydia*, aiming to induce mucosal immunity (75, 76); for example, an intranasal adenovirus vaccine generated tissue-resident T cells that protected against genital Chlamydia infection (77).

Viral vectors offer several advantages. They inherently stimulate innate immunity (through viral proteins and DNA), often obviating the need for external adjuvants (78). They can mimic natural infection routes – for instance, an intranasal adenovirus induces respiratory IgA and lung-resident T cells, which could be ideal for respiratory bacterial pathogens. Many vectors can carry large or multiple genes (poxviruses in particular have high payload capacity), enabling multivalent designs (79). However, there are challenges. Pre-existing immunity to the vector can blunt vaccine efficacy: many people have antibodies against common human adenoviruses (like Ad5), which may neutralize the vector on administration (80). Even novel vectors will induce anti-vector immunity after one dose, complicating boosters. Approaches to mitigate this include using rare or non-human adenovirus serotypes, or heterologous prime-boost with different vectors. Safety must also be monitored: while non-replicating vectors are generally safe, rare adverse events (e.g. clotting issues observed with some adenoviral COVID-19 vaccines) underscore the need for vigilance.

In summary, viral vector vaccines are a versatile and powerful platform. They excel at inducing both T-cell and antibody immunity, often with single-dose administration. Proven successes in TB research and other fields underscore their potential against AMR pathogens. Given growing regulatory experience and public acceptance (post-2020), viral vectors will continue to be a key tool in next-generation vaccine design (36).

## Perspectives and conclusion

Next-generation vaccine platforms provide a realistic path to bend the AMR curve by preventing the hardest infections before antibiotics are needed. mRNA stands out for speed, multivalency, and balanced humoral–cellular immunity; saRNA adds powerful dose-sparing for rapid, large-scale responses; DNA contributes stability, cost-efficiency, and T-cell-priming flexibility; viral vectors excel at potent Th1/CD8^+^ priming, especially for intracellular bacteria; nanoparticles maximize B-cell activation and adjuvanticity while enabling mucosal targeting. Rather than choosing a single “winner,” future programs should integrate platforms in heterologous prime–boost regimens tailored to pathogen biology (e.g., vector or DNA prime for TB T-cell imprinting → mRNA/NP boost for breadth; mRNA prime for ESKAPE toxoids → NP boost to deepen affinity maturation).

Three accelerants can shorten time-to-impact. First, AI-enabled antigen discovery that merges reverse vaccinology with structure-aware epitope mapping and pathogen population genomics will raise the hit rate for conserved, protective antigens and enable rational multivalency. Second, mucosal delivery innovations—thermostable LNPs, inhalable or intranasal formulations, and mucoadhesive nanoparticles—are essential for respiratory and gut pathogens where local IgA and tissue-resident T cells determine outcomes. Third, regulatory and financing pathways adapted from pandemic responses (rolling review, platform “master files,” delinked pull incentives, and advance market commitments) can de-risk late-stage trials for AMR vaccines and secure equitable access, particularly in low- and middle- income countries (LMICs), where the burden of bacterial multi-drug resistance—in terms of morbidity, mortality, and health-system impact—is highest.

Key uncertainties remain: defining immune correlates for each disease; optimizing the durability of vaccine-induced immune protection (e.g., sustained neutralizing antibody titers and long-lived memory B/T cells) without excessive reactogenicity; validating manufacturing at scale for newer materials; and designing pragmatic, event-driven efficacy trials in settings with evolving standards of care. Mitigation strategies include standardized functional assays (e.g., opsonophagocytic killing, toxin neutralization), systems-immunology endpoints, and adaptive trial designs embedded in surveillance networks.

In sum, mRNA and allied platforms have moved bacterial vaccinology from aspiration to actionable pipelines. With judicious antigen selection, rational combinations, and attention to access and implementation, next-generation vaccines can become central to AMR control—protecting high-risk patients, reducing antibiotic exposure, and preserving the efficacy of existing drugs. The field now needs coordinated investment to convert strong preclinical signals and early human data into licensure-quality evidence and scalable products. If we sustain the scientific, regulatory, and financing momentum established during COVID-19, a first wave of effective bacterial vaccines built on these platforms is a credible near-term goal.
